# Origins of cancer symposium report: beyond the tumor cell

**Published:** 2014-11

**Authors:** Andrew M. Howard, Eric A. Nollet, Nikki M. Thellman

**Affiliations:** ^1^ Laboratory of Cell Structure and Signal Integration, Van Andel Research Institute, Grand Rapids, MI, USA; ^2^ Laboratory of Integrin Signaling and Tumorigenesis, Van Andel Research Institute, Grand Rapids, MI, USA; ^3^ Laboratory of Transcriptional Regulation, Van Andel Research Institute, Grand Rapids, MI, USA

**Keywords:** Cancer, extracellular matrix, immunology, symposium, review

## Abstract

The Origins of Cancer Symposium is a meeting organized by graduate students at the Van Andel Research Institute and serves as a forum for focused discussion on factors that contribute to the etiology of cancer. The theme for the fifth annual Origins of Cancer held on July 11th, 2014 was Beyond the Tumor Cell, which focused on the complex influences coming from the environment in which the cancer cell exists. Here we report on the meeting proceedings and briefly discuss the far-reaching implications.

On July 11, 2014, scientists, clinical oncologists, and students from across the region convened for the fifth annual Origins of Cancer Symposium at the Van Andel Research Institute in Grand Rapids, MI. An adaptation of the original Oncogene meetings established by the Foundation for Advanced Cancer Studies in the 1980s, this one-day symposium was organized by senior Ph.D. students as part of their professional development training at the Van Andel Institute Graduate School [1-3]. The symposium showcased eight world-renowned scientists who discussed their novel findings related to this year's theme, *Beyond the Tumor Cell*, which focused on the complex influences coming from the environment in which the cancer cell exists.

The symposium began with talks highlighting the role of systemic inflammation in both health and disease. In his talk on inflammatory mediators, immune modulation and cancer progression, Raymond Dubois (The Biodesign Institute at Arizona State University) introduced the role of inflammation in cancer focusing on how NSAIDs are used to treat colorectal cancer. He outlined how anti-inflammatory therapy such as aspirin or celecoxib can be used to prevent progression in preclinical models of colon cancer [4-6]. Dr. Dubois described the mechanism by which immune cells are recruited to the tumor microenvironment and showed that blocking CXCR2 subverted tumor-induced immunosuppression and slowed cancer progression [7].

Susan Erdman (Massachusetts Institute of Technology) expanded the scope of inflammation and proposed that healthful longevity relies on microbial homeostasis, which properly conditions the immune system. In her talk about gut microbes and the cancer macroenvironment, Dr. Erdman suggested that early *in utero*, beneficial environmental microbes stimulate integrated immune and neuroendocrine factors throughout the body [8, 9]. Dr. Erdman focused on beneficial gut microbes and proposed that modulation of regulatory T-lymphocyte phenotypes through all stages of life may determine the fate of pre-neoplastic lesions [10]. She discussed microbial engineering strategies using food-grade bacteria as a biologically safe and efficient adjunctive approach to treating or preventing cancer progression.

Johanna Joyce (Memorial Sloan-Kettering Cancer Center) described her recent and exciting work targeting innate immunity in glioblastoma. After determining a strong correlation between tumor-associated macrophages and tumor progression, metastasis, and therapeutic resistance in her earlier work, the Joyce Lab has systematically proved that the colony stimulating factor-1 receptor is a viable therapeutic target in preclinical models of cancer, especially glioblastoma [11, 12]. Therapies based on this target were able to prevent glioma formation, shrink established tumors, and extend overall life span by reeducating macrophages and microglia in the tumor microenvironment from a tumor-promoting phenotype to a tumor-killing phenotype [13].

Drew Pardoll (Sidney Kimmel Comprehensive Cancer Center) highlighted his work on immunotherapy in his lecture about adaptive crosstalk between cancer and the immune system. He told the audience that immune checkpoint ligands and their receptors block the anti-tumor activity of cytotoxic T-cells in spite of tumor-specific immunity. These ligands are up-regulated in cancer and prevent cytotoxic T-cells from infiltrating the tumor. In support of this, he showed that patients treated with checkpoint blockade therapies have shown significant and persistent responses for even refractory melanomas [14, 15]. Additionally, preclinical models have shown that combinations of anti-checkpoint therapies are even more efficacious than monotherapies [16].

Shifting focus to the extracellular matrix (ECM) surrounding cancer cells, the symposium's afternoon speakers challenged the audience to continue to look beyond the intrinsic causes of cancer. Zena Werb (University of California, San Francisco) talked about the insights into cancer she gained by studying ECM proteolysis during development. Her studies of normal breast-ductal cells invading local tissue during development have led Dr. Werb to identify key factors involved in breast tumor cell invasion, such as the metastasis suppressor GATA3. She showed how GATA3 regulates miR29b expression, which in turn alters the expression of proteins that regulate angiogenesis and collagen proteolysis. Loss of GATA3 causes microenvironment remodeling that favors metastasis [17, 18].

In his lecture, Andrei Seluanov (University of Rochester) described his research on a collection of atypical rodent models that led to his discovery of novel anticancer mechanisms in long-lived mole rat species [19]. He demonstrated that adult naked mole rat fibroblasts undergo strong contact inhibition at less confluent numbers than mice or human fibroblasts [20]. The early contact inhibition is caused by a mutation in hyaluronic acid synthase II that allows the cells to autonomously produce large amounts of long-chain hyaluronic acid. Breaking down the hyaluronic acid with hyaluronase eliminates the early contact inhibition seen in culture and allows the cells to be transformed, which opens up the possibility of using these inhibitors therapeutically.

Patrick Moore (University of Pittsburgh Cancer Institute) shifted the focus to microbes in cancer development and progression in his talk about Merkel cell polyomavirus (MCV) and Merkel cell carcinoma. There have been only seven well-established and characterized tumor viruses, and none of the seven are similar. The Moore-Chang laboratory discovered MCV as the causative agent of Merkel cell carcinomas using a novel technique they developed called digital transcriptome subtraction [21]. MCV causes an asymptomatic infection of the skin and is actually very common, with 80% of those over 50 years of age having been exposed. In just four years, the molecular dissection of how this common virus causes Merkel cell carcinomas has led to new diagnostics and clinical trials [22]. Moore left the audience with the intriguing suggestion that, like the human microbiome, a human “virome” likely has both beneficial and disease-causing potentials that are worth exploring. With new technologies like digital transcriptome subtraction, more molecular success stories are likely.

Crislyn D'Souza-Schorey (University of Notre Dame) switched the focus back to the ECM with an intriguing story about how cancer cells release microvesicles to modify the ECM for cancer cell invasion [23]. Her talk emphasized that it is not just the microenvironment itself that has influence on the tumor, but that tumor cells are directly communicating with both the immediate environment and perhaps distant sites. She emphasized that the cargo within microvesicles (proteins, RNA, microRNA, and/or DNA) is selectively sorted and not randomly “pinched” into the microenvironment [24]. A regulated invasive mechanism of cancer that involves released particles has huge implications for biomarkers clinically. By detecting a microvesicle signature, doctors might enhance diagnostic and prognostic tools for specific cancers [25].

In selecting this year's theme, our objective was to focus discussion on tumor cell extrinsic factors that promote cancer. We wanted to learn about the ways the tumor stroma and the immune system interact with tumor cells to influence cancer progression. What crystallized for us was the astounding concept that a tumor cell has no true autonomy; it is functionally integrated into and actively communicating with its host environment. And what happens to that host environment, locally or distantly in space and time, can profoundly influence disease progression. Dr. DuBois emphasized it best in stating, “Focus on the tumor microenvironment is critical now… . Cells interact in ways you can't understand by studying the tumor alone.” This new concept raises many interesting questions on the origins of cancer that we look forward to answering as we embark on our own careers in science.

## References

[1] Burgenske D, Valkenburg K (2013). Origins of Cancer Symposium Report. Genes & Cancer.

[2] Duesbery NS, MacKeigan JP (2008). Origins of cancer. Oncogene.

[3] Hunter T, Simon J (2007). A not so brief history of the Oncogene Meeting and its cartoons. Oncogene.

[4] Wang D, DuBois RN (2013). The role of anti-inflammatory drugs in colorectal cancer. Annu Rev Med.

[5] Tsujii M, DuBois RN (1995). Alterations in cellular adhesion and apoptosis in epithelial cells overexpressing prostaglandin endoperoxide synthase 2. Cell.

[6] Wang D, Wang H, Shi Q, Katkuri S, Walhi W, Desvergne B, Das SK, Dey SK, DuBois RN (2004). Prostaglandin E(2) promotes colorectal adenoma growth via transactivation of the nuclear peroxisome proliferator-activated receptor delta. Cancer Cell.

[7] Katoh H, Wang D, Daikoku T, Sun H, Dey SK, Dubois RN (2013). CXCR2-expressing myeloid-derived suppressor cells are essential to promote colitis-associated tumorigenesis. Cancer Cell.

[8] Levkovich T, Poutahidis T, Smillie C, Varian BJ, Ibrahim YM, Lakritz JR, Alm EJ, Erdman SE (2013). Probiotic bacteria induce a ‘glow of health’. PLoS One.

[9] Lee CW, Rao VP, Rogers AB, Ge Z, Erdman SE, Whary MT, Fox JG (2007). Wild-type and interleukin-10-deficient regulatory T cells reduce effector T-cell-mediated gastroduodenitis in Rag2−/−mice, but only wild-type regulatory T cells suppress Helicobacter pylori gastritis. Infect Immun.

[10] Poutahidis T, Kleinewietfeld M, Erdman SE (2014). Gut microbiota and the paradox of cancer immunotherapy. Front Immunol.

[11] Shree T, Olson OC, Elie BT, Kester JC, Garfall AL, Simpson K, Bell-McGuinn KM, Zabor EC, Brogi E, Joyce JA (2011). Macrophages and cathepsin proteases blunt chemotherapeutic response in breast cancer. Genes Dev.

[12] Pyonteck SM, Gadea BB, Wang HW, Gocheva V, Hunter KE, Tang LH, Joyce JA (2012). Deficiency of the macrophage growth factor CSF-1 disrupts pancreatic neuroendocrine tumor development. Oncogene.

[13] Pyonteck SM, Akkari L, Schuhmacher AJ, Bowman RL, Sevenich L, Quail DF, Olson OC, Quick ML, Huse JT, Teijeiro V, Setty M, Leslie CS, Oei Y, Pedraza A, Zhang J, Brennan CW (2013). CSF-1R inhibition alters macrophage polarization and blocks glioma progression. Nat Med.

[14] Pardoll DM (2012). Immunology beats cancer: a blueprint for successful translation. Nat Immunol.

[15] Pardoll DM (2012). The blockade of immune checkpoints in cancer immunotherapy. Nat Rev Cancer.

[16] Brahmer JR, Pardoll DM (2013). Immune checkpoint inhibitors: making immunotherapy a reality for the treatment of lung cancer. Cancer Immunol Res.

[17] Chou J, Lin JH, Brenot A, Kim JW, Provot S, Werb Z (2013). GATA3 suppresses metastasis and modulates the tumour microenvironment by regulating microRNA-29b expression. Nat Cell Biol.

[18] Chou J, Shahi P, Werb Z (2013). microRNA-mediated regulation of the tumor microenvironment. Cell Cycle.

[19] Gorbunova V, Seluanov A, Zhang Z, Gladyshev VN, Vijg J (2014). Comparative genetics of longevity and cancer: insights from long-lived rodents. Nat Rev Genet.

[20] Tian X, Azpurua J, Hine C, Vaidya A, Myakishev-Rempel M, Ablaeva J, Mao Z, Nevo E, Gorbunova V, Seluanov A (2013). High-molecular-mass hyaluronan mediates the cancer resistance of the naked mole rat. Nature.

[21] Feng H, Taylor JL, Benos PV, Newton R, Waddell K, Lucas SB, Chang Y, Moore PS (2007). Human transcriptome subtraction by using short sequence tags to search for tumor viruses in conjunctival carcinoma. J Virol.

[22] Arora R, Chang Y, Moore PS (2012). MCV and Merkel cell carcinoma: a molecular success story. Curr Opin Virol.

[23] Muralidharan-Chari V, Clancy JW, Sedgwick A, D'Souza-Schorey C (2010). Microvesicles: mediators of extracellular communication during cancer progression. J Cell Sci.

[24] Muralidharan-Chari V, Clancy J, Plou C, Romao M, Chavrier P, Raposo G, D'Souza-Schorey C (2009). ARF6-regulated shedding of tumor cell-derived plasma membrane microvesicles. Curr Biol.

[25] D'Souza-Schorey C, Clancy JW (2012). Tumor-derived microvesicles: shedding light on novel microenvironment modulators and prospective cancer biomarkers. Genes Dev.

